# New Ways of Working? A Rapid Exploration of Emerging Evidence Regarding the Care of Older People during COVID19

**DOI:** 10.3390/ijerph17186442

**Published:** 2020-09-04

**Authors:** Éidín Ní Shé, Deirdre O’Donnell, Marie O’Shea, Diarmuid Stokes

**Affiliations:** 1 School of Nursing, Midwifery and Health Systems, University College Dublin, Belfield 4 Dublin, Ireland; eidin.nishe@ucd.ie (É.N.S.); marie.oshea@ucd.ie (M.O.); 2 Liaison Librarian for Health and Science, University College Dublin, Belfield 4 Dublin, Ireland; diarmuid.stokes@ucd.ie

**Keywords:** integrated care, older people, Covid-19, new ways of working, health and social care, teamwork, social media

## Abstract

Health and social care staff have had to quickly adapt, respond and improve teamwork, as a response to the COVID-19 pandemic. Our objective was to rapidly summarize the emerging evidence of new ways of working in the care of older people during this period. We conducted an exploration of the emerging evidence within the timeframe of 1 March 2020 to 11 May 2020. To capture a broad perspective, we undertook thematic analysis of Twitter data which was extracted through a broad search for new ways of working in health and social care. For a more in-depth focus on the health and social care of older people, we undertook a systematic scoping of newspapers using the Nexis UK database. We undertook a validation workshop with members of the interprofessional working group of the Irish National Integrated Care Programme for Older People, and with researchers. A total of 317 tweets were extracted related to six new ways of working. There was evidence of using telehealth to provide ongoing care to patients; interprofessional work; team meetings using online platforms; trust and collaboration within teams; as well as teams feeling empowered to change at a local level. 34 newspaper articles were extracted related to new ways of working in the care of older people, originating in England (*n* = 17), Wales (*n* = 6), Scotland (*n* = 6), Ireland (*n* = 4) and Germany (*n* = 1). Four main themes were captured that focused on role expansion, innovations in communication, environmental restructuring and enablement. The results of this exploration of emerging evidence show that health and social care teams can transform very rapidly. Much of the change was based on goodwill as a response to the pandemic. Further analysis of empirical evidence of changing practices should include the perspectives of older people and should capture the resources needed to sustain innovations, as well as evaluate gaps in service provision.

## 1. Introduction

On the 11 March 2020, the World Health Organisation declared that severe acute respiratory syndrome secondary to the novel coronavirus disease (SARS-COV-2) was a worldwide pandemic [1]. Currently (19 August 2020) 1,962,958 cases of COVID-19 have been reported in the European Union and the United Kingdom, including 179,963 deaths [2]. Research into a potential treatment and vaccine development is ongoing but caution has been urged that this will take time [3]. As a response to the pandemic, sweeping changes have occurred in health and social care systems to mitigate the virus [4,5,6]. The emerging research has found that Covid-19 disproportionately affects older people. Older people are more likely to require hospital admission and they are most likely to die from COVID-19 infection [7,8,9,10,11,12]. As a response to COVID-19, Ireland’s National Public Health Emergency Team (NPHET) followed the guidance of the European Centre for Disease Prevention and Control who recommended several measures. These included the closure of non-essential services and travel on the 27 March 2020 to limit human to human transmission [13]. As of the 19 August 2020, Ireland has had 27,499 confirmed cases including 1738 deaths [14]. Following trends in other countries, people over the age of 70 were deemed as particularly vulnerable to COVID-19. A specific recommendation was made that people over 70 should ‘cocoon’ at home to reduce face-to-face interaction with other people [11].

Across the globe, frontline health and social care staff experienced exceptional demands such as dealing with high mortality, rationing of personal protective equipment and ethical dilemmas involving rationing access to ventilators and other essential supplies [15]. Since the restrictions were introduced in Ireland in March 2020, health and social care staff have had to adapt and respond to the COVID-19 pandemic. The literature notes that teamwork during the pandemic has become both essential and challenging [16,17]. Capturing evidence of changes that have occurred within teamwork during this critical responsive period is important in developing an understanding of these new ways of working. This critical understanding will contribute knowledge of team dynamics in healthcare settings and may identify new ways of working which are beneficial for inter-professional collaboration and have the potential to be sustained [18,19]. To commence the capture of this evidence, we decided to undertake a scoping review of the available emerging evidence during COVID-19 [20]. Scoping reviews are particularly useful to capture emerging evidence, when it is unclear which more specific questions can be posed, for evidence synthesis [21].

## 2. Methods

Two complementarity approaches were undertaken to enable us to characterize and map the current evidence of new ways of working in the care of older people during COVID-19. An explorative descriptive study design was implemented using a modified version of Arksey and O’Malley’s framework [22] that included identifying the research focus, identifying databases to search, generating inclusion/exclusion criteria, study screening and extraction, and external validation.

### 2.1. Identifying the Research Focus

In April 2020 we held three video consultations with our college librarian (DS) during which we reviewed potential questions and scope before agreement on refinement. For this exploration of new ways of working in the health and social care of older people during COVID-19, we wanted to capture some of the emerging evidence from two sources: Twitter and newspapers. For breadth and without any geographical restrictions we decided to search Twitter for data which described new ways of working in health and social care generally, without limiting this to the care of older people. For a more in-depth focus on the care of older people we decided to capture newspaper coverage from Europe, where many countries were experiencing a peak of COVID19.

### 2.2. Identifying Databases

The study was undertaken in May 2020. We followed two parallel approaches to identify relevant emerging evidence pertaining to a broad understanding of new ways of working (breadth) as well as a more in-depth view of the context of older people’s health and social care (depth).

#### 2.2.1. Breadth

An analysis of posts on Twitter was conducted to collect information about new ways of working within health and social care during the period of the COVID-19 pandemic. Twitter is a social media microblogging platform that provides the user with 280 character ‘tweets’ that may consist of images, text, and links [23]. Approximately 500 million tweets are sent per day and Twitter has 316 million active users [24]. When a person registers to a Twitter account other users can follow them and see their tweets. Previous work has found that analysis of the content on social media has become a valuable source of information for health researchers [25,26,27] The information provides researchers with publicly available data that would not be accessible using more traditional methods for data collection [28]. Previous work has highlighted that peer interaction on social networks such as Twitter can contribute to policy development in health and social care [28,29].

We used the Twitter advanced search function to inform our scoping review [https://twitter.com/search-advanced?lang=en]. As there was no generic hashtag, we decided to search broadly using new ways of working (Table 1).

#### 2.2.2. Depth

Following some testing of keywords by the college librarian in April 2020 using several academic journal databases, it became clear that depth would not be achieved from searching relevant academic articles. It was agreed to undertake a systematic approach to retrieve newspaper articles which described new ways of working in the health and social care of older people as a result of the COVID 19 pandemic. Previous scoping reviews have included newspapers to capture emerging evidence [21,30]. The search was conducted in the Nexis UK database which is a curated archive of the UK and Ireland’s national and regional newspapers as well as international newspapers and newswires. The timeframe for the search was limited to articles published between 1 March 2020 and 11 May 2020.

The search terms used, either singularly or in combinations were: (“Older People” OR Elder* OR Senior* OR Pensioner* OR “Over 65′s” OR Cocooner* OR Geriatric* OR Resident* OR OAP OR Aged OR Grandparent OR Centenarian* OR Retiree* OR “Retired person”) AND (HCP* OR “Healthcare Professional*” OR Doctor* OR Consultant* OR Intern* OR “Senior House Officer*” OR Registrar* OR Attending OR Physician* OR “General practitioner*” OR medic OR Nurse* OR PHN OR RGN OR “Allied Health” OR “Occupational Therapist*” OR OTs OR “Speech and language therapist*” OR SLTs OR Dietitian* OR Physiotherapist* OR PTs OR “Social worker*” OR HSCPs OR “Health and Social Care Professional*” OR Paramedic* OR “Health care assistant*” OR HCAs OR Carers OR “home help” OR “Home visit*” OR care) AND (Covid-19 OR Coronavirus OR MERS-CoV OR “2019 nCoV” OR 2019nCoV OR “COVID 19” OR COVID-19 OR “SARS CoV-2” OR “SARS-CoV” OR “2019-nCoV” OR “SARS-CoV-2”) AND (Inter-disciplinary OR interdisciplinary OR Inter-professional* OR Interprofessional* OR Team* OR Collaboration* OR “Collective leadership”).

### 2.3. Inclusion and Exclusion Criteria

The authors participated in multiple video meetings to determine the criteria for inclusion of articles or tweets. For the Twitter search, it was agreed to include all tweets in English mentioning ‘COVID-19′ and “new ways of working’ within ‘health’ and ‘social care’.” No geographic exclusion was placed on the search. We did not focus on any specific health and social care discipline. Our focus was to capture the breadth of tweets related to any new ways of working occurring at the time of the search. Excluded tweets would include those not specifically mentioning or relevant to health and social care. The criteria for inclusion of newspapers in the review were ‘English language’ (or translations). We searched for newspaper articles published in Europe under the subject category of ‘medicine and health’ and focusing on the healthcare industry. We excluded articles that did not focus on the care of older people or where the primary focus of the article was not the care of the older person. Articles were also excluded if they did not describe changes in the work practices and teamwork of health and social care professionals as a response to the COVID 19 pandemic.

### 2.4. Screening and Extraction

Relevant health and social care tweets were collected by one reviewer (É.N.S.) using the NCapture tool for NVivo. NCapture is a Chrome web browser extension for NVivo12 (QSR International, Doncaster, Australia) that can be used to create a chronological dataset or ‘batch’ of tweets, working backwards from the time of the ‘capture’ [28]. We used descriptive statistics to describe the sample and thematic analysis for the resulting qualitative data set using NVivo nodes. Thematic analysis is a process of identifying patterns or themes within qualitative data [31]. Using the Twitter advanced search option thousands of tweets were retrieved which mentioned ‘news ways of working’, covering areas such as education, health, work, family and personal tweets (supplementary file 1-Table S1). The initial codes for the extracted tweets were based on the specified research question and identified tweets which were most relevant to health and social care and COVID-19. Following a review and discussion by team members (É.N.S., M.O. and D.O.), a final set of codes were agreed. The extracted tweets were divided between the two members and codes were developed and discussed by the team over video discussion. Following best practice guidance, the usernames are not presented in the results section [28,32].

**Figure 1:**
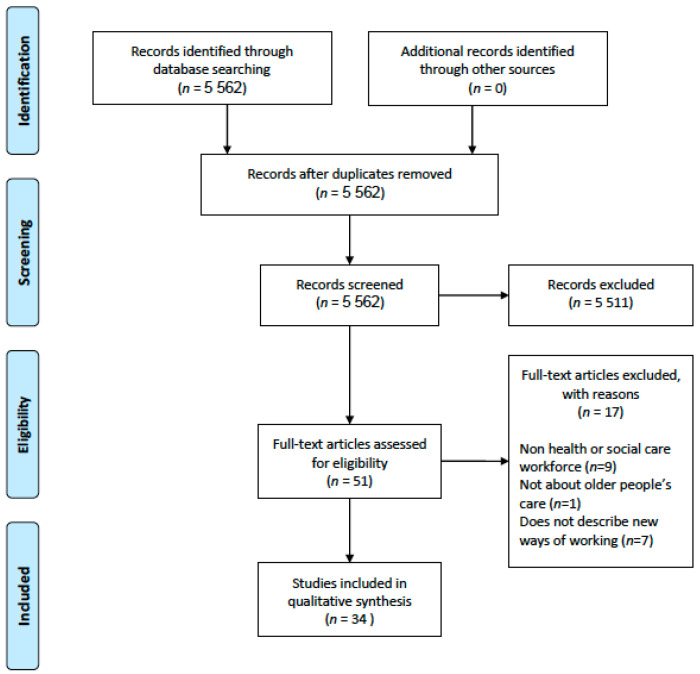
PRISMA Flow Diagram.

The newspaper search was conducted on 12 May 2020 yielding a total of 5562 articles for full-text screening. Two reviewers (É.N.S., M.O.) screened the full-text articles based on eligibility criteria with a third reviewer acting as moderator (D.O.). A total of 51 articles were identified in the initial review. A further full-text screening conducted by all three reviewers yielded a final 34 articles identified as eligible for extraction (see Figure 1).

An extraction template was created (supplementary file 2, Table S2–S10). Three researchers extracted the articles noting the country of origin, the healthcare setting described, and the specific health and social care professionals involved. The extraction criteria included two types of innovations: changes in the roles and behaviours of individuals and teams as well as innovations in the organisation of how healthcare resources, structures and contexts are governed and managed in the care of older people. The social processes underlying the innovation were also noted with reference to the human and social resources, competencies, motivations, reasoning and interrelationships being described. Finally, the extraction of information from the articles identified ‘new ways of working’ in providing health and social care to older people during the pandemic (context).

### 2.5. Validation

After our data extraction, we extended an invitation to attend a validation workshop to members of the interprofessional working group of the Irish National Integrated Care Programme for Older People and with researchers in the University College Dublin Centre for Interdisciplinary Research Education and Innovation in Health Systems. Validation and feedback were undertaken via videoconference on the 2 July 2020 with 11 participants. Research team members É.N.S., D.O. and M.O. provided a summary of the key findings. Feedback on the findings was sought. Specifically, we asked if our findings were consistent with their own experiences of working in the care of older people during COVID-19. Further analyses suggested by the experts were incorporated into our final manuscript, specifically in the discussion section.

## 3. Results

### 3.1. Twitter: New Ways of Working in Health and Social Care during COVID-19

A total of 317 tweets were extracted relating to six new ways of working in health and social care during COVID-19 between 1 March 2020 and 11 May 2020. These were:

1. Using telehealth and or phone consultations to provide ongoing care to patients
2. Interprofessional work
3. Team meetings using online platforms
4. Trust and collaboration within teams
5. Sharing information and a clear feedback loop between teams
6. Teams felt empowered to change at a local level

The tweet themes are shown in Table 2 with supporting examples.

A majority of the tweets extracted (*n* = 111) were themed under teams being empowered at the local level to change. There was clear evidence of teams being able to implement changes quickly at a local level. Evidence of pride expressed by health and social care workers in what they had achieved, was clear, with a desire that the changes should be sustained following the peak of the pandemic. Interprofessional work (*n* = 79) was the second most frequent theme in the twitter data that described ‘new ways of working’ in health and social care. The tweets relayed how team members had taken on new roles and that there had been a removal of siloed and hierarchical structures during COVID-19. There was clear evidence of new ways of working in the delivery of care with the introduction of telehealth and phone consultations (*n* = 71). The tweets cited that this initiative was something health and social care teams wanted to retain after the peak of the pandemic. How teams were engaging with each other also appeared under new ways of working (*n* = 22). Various online platforms were highlighted that had assisted team engagement, team huddles and debriefs across various sites. There was evidence of trust and collaboration amongst teams, enabled by the COVID-19 pandemic (*n* = 18). Finally, tweets (*n* = 16) showcased how teams were sharing information amongst and between teams and there was also evidence of feedback loops being created right across the health system with additional information provided.

### 3.2. Newspaper Articles: A focus on New Ways of Working in the Care of Older People during COVID-19

**Figure 2:**
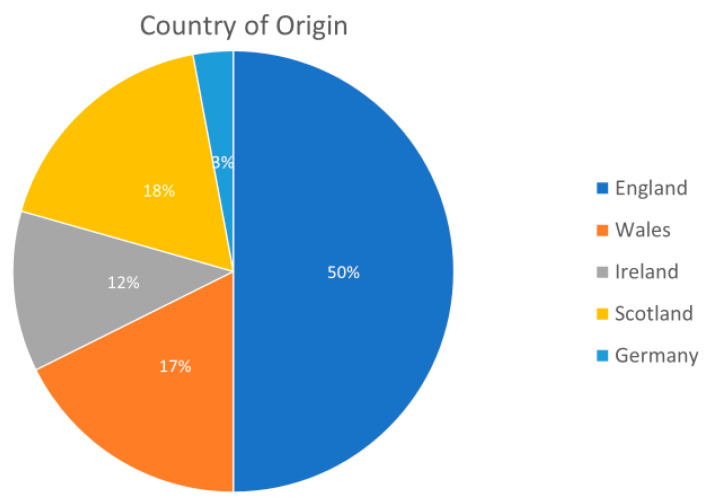
Pie chart showing the distribution of extracted articles by country of origin (*n* = 34).

A total of 34 articles were identified for extraction originating in England (*n* = 17), Wales (*n* = 6), Scotland (*n* = 6), Ireland (*n* = 4) and Germany (*n* = 1). (Figure 2).

**Figure 3:**
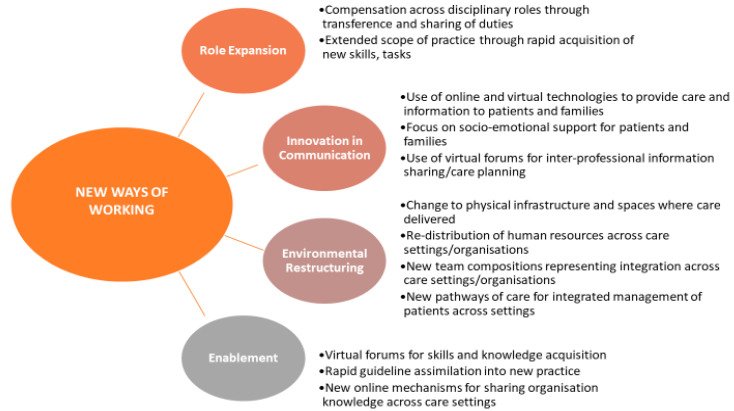
Summary of Key Themes for New Ways of Working Described in Newspaper Articles (*n* = 34).

The articles focused on healthcare settings across the care continuum for older people including community care, acute hospital care, and residential care and rehabilitation settings. Figure 3 provides a summary of the key themes described in the 34 newspaper articles.

The 34 newspapers articles focused on new ways of working in the care of older people and provided evidence for an in-depth understanding of changing practices.

Role expansion was captured in two ways from newspaper coverage. There was evidence of staff transferring and expanding from their normal duties [33,34,35,36,37,38]. One example saw cleaning staff stepping in as carers in a hospital ward in Wales with another example of council workers in England being redeployed as home carers to support older people in the community [39,40,41,42,43,44]. Other examples noted an expansion in the scope of the roles of health and social care staff [45,46]. One such initiative involved General Practitioners (GPs) and hospital physicians in Wales distributing iPads to care home residents to enable telehealth consultations [47]. Another story from Wales saw hospital physicians collaborating with GPs and community teams to deliver care directly in the home setting [48].

A story from Ireland mapped the rapid transformations that occurred between general practitioners and community pharmacists [49]. By working together they reorganised services to provide care to older patients. This reorganisation included arranging deliveries of prescriptions for older people who were cocooning and pharmacists changing their protocols to allow for electronic prescriptions from GP’s [49].

The retrieved newspaper articles described significant innovation in communication [49,50,51,52,53,54,55,56]. This included the use of on-line tools to support service provision to older people as well as facilitating communication with family members [57,58,59]. Coverage also highlighted significant uptake by healthcare staff of virtual forums and websites to support training, interprofessional care-planning and information sharing [50,56].

Healthcare professionals were described in the newspaper articles as demonstrating strong communication skills in providing emotional support to older people to compensate for the physical distancing. One story from a nursing home in Scotland, for example, outlined how staff provided ongoing verbal emotional support to residents in the absence of physical contact [51]. Changes to the social and physical infrastructure where care was delivered were captured in the retrieved articles [42,46,60,61,62]. One story from Germany outlined how a nursing home created three separate zones for residents based on their triage status [61]. Newspapers also captured the redistribution of staff across care settings and the establishment of new integrated care teams [40,42,63,64]. There was also evidence of new pathways of care being developed to protect older people transitioning between services [49,52,65,66]. A story from England for, example, mapped how a front line social care team from a local council worked within hospitals to expedite the discharge of older people to appropriate community settings [67].

There was evidence within the articles of rapid acquisition of knowledge and training regarding the clinical management of Covid-19. One exemplar from England found care home staff working across different settings meeting virtually to collaborate, share knowledge and support each other [57]. The rapid development and adoption of clinical guidelines were also highlighted. An Irish exemplar noted how staff in a particular setting had to rapidly respond to guidelines around personal protective equipment [50]. The use of online resources was the mechanism used by teams to receive education and training and to acquire information rapidly [58,67].

### 3.3. Validation Workshop

During the validation workshop much of what was captured in the review corresponded with the experiences of the health and social care workers who participated. Attendees outlined how barriers were removed quickly for them and they provided examples of work they had done to reconfigure services and embed telemedicine into practice with older patients. One attendee noted how a local government sports partnership had worked with her team to develop exercise classes for older people who were at home. Exercise classes were loaded onto a tablet and they developed simple user instructions where internet access was not required. Other participants noted how health and social care professionals developed webinars and video conferences to share learning with nursing home staff across public and private providers at the height of the COVID-19 crisis. Workshop attendees noted how hierarchies and seniority in roles disappeared as people worked together on a common goal. Concern was raised on service provision gaps that had occurred due to redeployment and new ways of working. Examples outlined how one redeployment had left a team without a social worker whilst community occupational therapists had been redeployed to do testing/contact tracing. The participants noted that further research and exploration of these gaps in service provision was required particularly where there was potential for deconditioning, increasing the risk of frailty and/or hospital admission. Other concerns expressed related to the psychological impact on staff and, significantly, burnout and staff retention.

## 4. Discussion

This exploratory study searched for evidence of new ways of working during the peak of the COVID-19 pandemic in Europe and in particular in Ireland and the United Kingdom. The Twitter exercise provided a broad overview of emerging evidence of new ways of working within health and social care teams. There was evidence of teams expressing excitement at being empowered at a local level to bring about changes. Tweets noted that much of the changes that have been introduced would normally have taken years to implement, such as the introduction of telehealth. Central to the success of the changes was teamwork and this aligns to the literature [16,17]. Added to this was evidence of interprofessional work, described as cooperative and boundary spanning and noted by one tweeter as having changed ‘beyond recognition’. Evidence of increased trust and collaboration was also seen locally. Tweets noted the pressures experienced by all, but strong relationships were key enablers to overcome these. There were many examples tweeted of health and social care teams using various online platforms to continue to meet and communicate with each other and include teams outside of their settings. The sharing of information across sites and within teams was done quickly and it was clear from the tweets that staff were able to feedback their perspectives.

The scoping of newspapers provided an opportunity to capture a more in-depth focus on health and social care teams working in the care of older people. The COVID-19 pandemic disproportionately affects older people [7,8,9]. The scoping review of newspapers found evidence of remarkable efforts by health and social care teams to ensure that older people remained COVID-19 free. Coverage noted how staff had moved into residential settings and many had expanded their roles. Examples highlighted how care homes and older person wards went to great efforts to ensure emotional support for older people to try to compensate for a lack of physical contact with the family. Examples included music on the wards, singing, and social initiatives. There was evidence of innovation in communication within teams, ensuring information was shared quickly across boundaries. This corresponds with the descriptions emerging from the synthesis of Twitter data. Healthcare teams worked together to understand new guidelines and collaborated to implement them. Significant coverage captured the environmental restructuring that occurred, both physical and social. One example described hospital teams stepping into new roles by undertaking home visits to support older people.

This exploratory study demonstrated that the health and social care system can transform very rapidly when presented with a single focus or threat. The context in which these changes have occurred is unprecedented. The level of risk for a potential second wave of COVID-19 is still unclear [1,2]. It is important to capture the changes that have occurred in this current wave of the pandemic to support the identification of new ways of working. Previous work has noted that social media sites such as Twitter can be used for real-time content analysis and knowledge translation research especially during a pandemic [68]. More recently, academic literature has noted the benefits of undertaking analysis of social media posts to understand the interprofessional experiences of clinicians during COVID-19 [69]. This was a clear finding in our review, in which tweets clearly expressed evidence of new ways of working and a desire to sustain these changes.

It is necessary to reflect upon both benefits as well as negative effects of practice changes and consider the potential to sustain innovations [18,19]. It should be stressed that the new ways of working occurred within a context of health and social care teams working way beyond their current roles. This was done with a significant degree of staff goodwill and commitment to their patients and colleagues in the face of an unprecedented threat to public health. Further research should capture the resources that are needed to support the sustaining of innovations that have occurred. Emerging academic literature focusing on the impact of COVID19 on health systems is capturing how healthcare staff are adapting and expanding their practices, thereby enabling the health system to respond to this public health emergency [70,71]. Sustaining positive changes, particularly those pertaining to inter-professional collaboration, communication and sharing of information, will require ongoing support and resourcing [72,73]. Diverse communication was a key enabler for health system preparation and responsiveness, identified in our exploratory study. The need for innovative communication, including tele-health, expedited referral pathways and information sharing within multidisciplinary health and social care teams and by senior leaders has also been identified in the emerging literature [74].

### Limitations

Our study does have some limitations. For this review, we searched Twitter and one newspaper database. Nexis UK database archives regional newspapers from the UK only, along with national papers more globally. A further limitation is our focus only on English language Twitter and newspaper publications. Future work should capture and synthesise the anticipated outputs from ongoing and emerging robust academic research describing and evaluating practice innovations and health systems responses to the pandemic. In particular, attention should focus on learning in other territories where the impact of Covid19 has increased such as Canada, the United States of America, Brazil and Australia. We are mindful that our Twitter and newspaper search looked for the positives of ‘new ways of working’ but was a useful exercise to capture real-time insight [69]. Through consultation with the inter-professional sub-group of the National Clinical Programme in Ireland, we were able to validate our review findings. This enables us to draw some conclusions from our work within the parameters of an initial exploratory study. Our validation identified the concerns of those directly working in the health system. These concerns should be explored in detail in further work. Notably missing from Twitter and the newspaper articles were the direct insights into these changes from older people themselves. Their exclusion is aligned to a broader decrease in patient and public participation in research and policy that has occurred during the pandemic [11,75]. Recognising this gap, organisations such as the British Geriatrics Society have called for the inclusion of older people in COVID-19 research [76]. Further work should prioritise older peoples’ perspectives. Following our validation workshop, further work should capture the impact of potential gaps which the response to COVID-19 has left in service provision and the composition of inter-professional teams.

## 5. Conclusions

To the best of our knowledge, this is the first exploratory study to collate new ways of working in the delivery of care for older people during the COVID-19 pandemic. The emerging evidence shows that it is older people who are disproportionately impacted. This exploration describes how health and social care teams transformed very rapidly. Much of the change was based on goodwill as a response to the CODID-19 pandemic. Further work should capture the resources and support needed to expand these new ways of working. Central to this is the involvement of older people themselves.

## Figures and Tables

**Table 1:** Twitter Advanced Search.

All of these Words	New Ways of Working
Language	English
Any of these Words	Health and Social Care OR Health or Social OR Care
Dates	1 March 2020 and the 11 May 2020

**Table 2:** Main Themes Supported by Tweets.

Theme	Tweets	Tweets Supporting the Theme
Using telehealth and/or phone consultations to provide ongoing care to patients	71	We will definitely keep this moving forward and continue to embrace the new ways of working. Primary care is now a blend of face to face and digital medicine. Safety first as always. Many of my patients happy to share video consultations but important to remember not everyone has tech still. 9 May 2020
		The Nurse Service is here to support any families caring for someone with dementia during this difficult and worrying time (new ways of working- telephone clinics, consultations, practical help, advice and support) supporting our community. 21 April 2020
		First #telehealth call with my youngest child today. In 20 mins he went from hiding and running from camera to smiling and waving. So grateful to parents for their flexibility and patience as we find new ways of working. #mySLTday. 20 March 2020
		Learning new ways of working during #COVID-19. Did ward round in psychiatry with registrar, nurse and OT in room with patient and consultant on Webex due to having to isolate. Patients coped quite well. 19 March 2020
Interprofessional Work	79	“It’s changed beyond recognition”—Many of our staff have had to find new ways of working, or take on new roles entirely, and the response has been brilliant. 1 April 2020
		I’m so impressed with the speed that our staff have implemented and adapted to new ways of working to provide therapeutic interventions during the barriers that face us in this challenging time. #Covid_19 #NHSheroes 20 March 2020
		Community spirit, Covid-19 shows the true strength of interdisciplinary cooperation and cross boundary working, no time for “me” or professional boundaries that are barriers to common good. New ways of working and long may they last! 14 March 2020
		Doctors and HCW are working together at all levels to prepare for an outbreak of #COVID-19 in the coming weeks. Whatever is required we will be there, delivering care. This may require redeployment and new ways of working, and we will do our best and our duty. 10 March 2020
Team meetings using online platforms	22	Working in new ways in our perinatal mental health team: Teams enables us to huddle with a virtual huddle board, & team drop-in at end of day: chance to think, connect and be ready for next day. + less emails and more conversations mean faster progress. Adapting positively. 6 April 2020
		It’s a strange time but look we did a virtual handover yesterday. Community nurses are used to mobile working and problem solving. 22 March 2020
		Teams testing out #Webex today. Checking in with our staff across primary care team, keeping ourselves up to date with new norms and new ways of working. 21 March 2020
		ED ACPs ENPs and Team SDEC evening get together in this new world, comes new ways of working, connecting and learning. 19 March 2020
Trust and collaboration within teams	18	Local relationships, trust & new ways of working at the heart of health & social care integration/wider service reform have been the bedrock of our ability to respond to C-19. They have to continue be the foundation of what we do next. 10 May 2020
		The last few weeks have brought challenges, gripes and niggles to say the least. However, they have also brought new and innovative ways of working with all different staff groups! Diversity and Inclusion have produced teamwork for a shared goal #CriticalCare 29 April 2020
		My colleagues (SHOs, SpRs, consultants) have all been amazing. We have changed to completely different ways of working - more weekends, nightshifts, new clinical challenges. Everyone has come on board and we’ve retained a really high morale despite the stress everyone is under. 18 April 2020
		We look for the positives at work. Things we have noticed are how quickly we can adapt to new ways of working. Clear channels for communication Even more #kindness from local community and between colleagues. 3 April 2020
Sharing information and a clear feedback loop between teams	16	Thank you from the leadership team to all our Older Adult Services teams in #Location- you’ve continued to work tirelessly to provide the best care possible & embrace new ways of working. Feedback has been really positive. Well done! #OurNHSPeople 29 April 2020
		We know that a lot of our teams are adjusting to new ways of working, so we’ve set up a Clinical Support line to provide mentoring and reassurance. 21 April 2020
		Due to #COVID-19 you may find yourself working in different ways, different settings or with new teams. Our guidance for support workers in health & social care include team working in rapidly changing environments, keeping a record of care & communication. 17 April 2020
		Social distancing means people are getting used to new ways of working. Today colleagues joined us to learn how they can work together & collaborate virtually: we look forward to seeing how you put your learning into action. 19 March 2020
Team felt empowered to change at a local level	111	We now have to look through a new lens as to how we deliver health services in Ireland. We need to retain some of our responses for #Covid-19 as they have proven good for the public. Working closer with GPs and unlocking the huge passion of staff are just two. 26 April 2020
		There have been countless innovations & new ways of working. Changes that might have taken years have been achieved in days in hospitals, mental health, GP & community services. As one leader said, we’re not going back to normal, we must embrace a new & very different future. 18 April 2020
		So many examples of great teamwork and leadership. Over the past few weeks - discharge pathways completely rewritten, different ways of working implemented and new teams formed. Very proud of everyone’s effort and resolve. 7 April 2020
		I have never known so much change happen so quickly. Honestly the NHS in a crisis is amazing, people working together all over the place and achieving so much and developing new ways of working. All with care and compassion for each other and the patients. 26 March 2020

## Supplementary Materials

The following are available online at https://www.mdpi.com/1660-4601/17/18/6442/s1. Supplementary file 1: The Twitter Advance Search Results. Supplementary file 2: Table S1: Newspaper Extraction Template, Supplementary file 3: Table S2–S10: Extraction for Retrieved Newspaper Articles (*N* = 34).
