# Supplementary material for: New Ways of Working? A Rapid Exploration of Emerging Evidence Regarding the Care of Older People during COVID19
Source: Int J Environ Res Public Health. 2020 Sep 4;17(18):6442. doi: 10.3390/ijerph17186442 (PMC7558069; doi:10.3390/ijerph17186442)
Supplement: Supplementary file 1 [file ijerph-17-06442-s001.pdf]

Supplementary file 1:
The Twitter Advanced Search Results:
https://twitter.com/search?f=live&q=New%20ways%20of%20working%20(%22Health%22%20OR%20%22Social%20OR%20Care%22%20OR%20Health%20OR%20and%20OR%20Social%20OR%20Care%22)%20until%3A2020-05-11%20since%3A2020-03-01&src=typed_query
Supplementary file 2:

Table S1. Newspaper Extraction Template.

**Title of article**
Source
Byline or Author(s)
Date of publication
Country of publication
Country of article focus
Health and social care personnel involved
Context for change (community, hospital, rehab or residential care)
Innovation (What did they do or change?)
Social processes (How or why did they do it? Motivations, reasonings, resources, competencies)
New ways of working (Outcome)

Supplementary file 3:

Table S2. Extraction Table for Retrieved Newspaper Articles (*N* = 34).

**Title of Article**	**"I Have Never Seen Such Devotion to Duty"**	**"It's an Honour to be the One Holding Their Hand"**	**"On the First Day I was Really Worried I Wouldn't Make it"**	**"The Panic was Huge, but we had to Work as a Team to Give Best Care"**
**Source**	South Wales Argus	Wales Online	The Argus	Irish Independent
**Byline or Author(s)**	Elizabeth Birt	Bethan Thomas	Samuel Brooke	Catherine Fegan
**Date of publication**	10.05.20	11.05.20	08.05.20	29.04.20
**Country of publication**	Wales	Wales	England	Ireland
**Country of article focus**	Wales	Wales	England	Ireland
**Health and social care personnel involved**	HSCPs (porters, domestic staff, ODPs, paramedics, physios, nurses and doctors)	Carers and nurses	HSCPs ('cleaners to senior consultants')	Nursing home staff (physicians, physical therapists, nurses and nursing assistants, social workers and community geriatrician).
**Context for change (community, hospital, rehab or residential care)**	Hospital	Residential (nursing home)	Hospital	Residential (nursing home)
**Innovation (What did they do or change?)**	Physical restructuring of space 'white boxes' and reorganisation of communication to reduce patient contact<br>Role expansion esp for cleaning staff—use of PPE, increased workload<br>Regular communication channels established between staff and patient and/or family including doctors ringing pts after discharge to check in, facilitating families to speak with patients and regularly ringing family during admission	Physical reorganisation of space to reduce physical contact 'red isolation zones'<br>Role expansion to work in the 'red zone' areas and increase working hours<br>Sleeping in the care home to limit exposure at home,<br>Nursing staff transfer from hospital to increase capacity<br>Sharing advice and info with other care home managers<br>Social restructuring to be with	Social reorganisation to improve communication and cooperation between staff and with staff and patient	Music on corridors, face-time virtual visits, visits to the gardens to compensate for visitor restrictions<br>Virtual staff meetings using video conferencing<br>Integration of acute system with the community 'public health' system for testing—using hospital staff and resources for swabbing and for the conduction of testing<br>Staff exposed through lack of

		dying patients and to open up virtual contact with patients and families.		access to PPE. Hospital staff provided training with regard to PPE<br>Staff encouraged to express concerns during daily ward rounds<br>Team of social workers 'manning phones' to keep contact with families allowing nurses to concentrate on patients (role adaptation and expansion)<br>Staff taking place of families as patients were dying—personalising their care and establishing virtual communication for family
**Social processes (How or why did they do it? Motivations, reasonings, resources, competencies)**	Commitment to their role and willingness to expand it to meet the challenge<br>Recognition of the importance of communication and finding ways to communicate without direct contact (patient notes, phone calls)	Interrelationships between carer/nurse and the patient—'taking the place of family'<br>Commitment to the role—willingness to expand it and take on more hours to meet the challenge<br>Recognition of the importance of social and emotional connection in the absence of physical contact<br>Willingness to share expertise and knowledge	Inter-professional relationships leading to good 'team spirit' rising to the challenge<br>Development of social relationship with patient—regularly 'checking in' to compensate for lack of physical contact	Integration of personnel and services to compensate for acute and community—reimagining 'the team'<br>Role expansion and compensation (social workers and nursing staff)<br>Commitment to social and emotional support for patients in the absence of physical contact
**New ways of working (Outcome)**	Role expansion and improved info sharing/communication	Role expansion, physical restructuring, social restructuring and improved info sharing/communication	Improved information sharing and communication	Integration of personnel and services across acute and community<br>Role expansion and inter-

				professional compensation<br>Social and emotional communication

Table S3. Extraction Table for Retrieved Newspaper Articles (*N* = 34) cont.

**Title of article**	**'Plan the Exit Now? I wouldn't have Time for that'; Emergency Clinics are being Set Up in Several Large Cities…**	**'We are Working Really Hard as a Care Community'**	**350 iPads to be Delivered to Care Homes and Hospitals Across North Wales…**	**A day in the Life of a Home Carer in Worchestershire during Coronavirus Pandemic**
**Source**	Die Welt	Carmarthen Journal	Denbighshire Free Press	Malvern Gazette
**Byline or Author(s)**	Nike Heinen	N/A	Arron Evans	Jemma Bufton
**Date of publication**	06.04.20	22.04.20	23.04.20	17.04.20
**Country of publication**	Germany	Wales	Wales	England
**Country of article focus**	Germany	Wales	Wales	England
**Health and social care personnel involved**	Doctors, fire department	Doctors, nurses, health care support workers, carers, assistants, transport workers, managers and volunteers	GPs and hospital physicans	Carers
**Context for change (community, hospital, rehab or residential care)**	Hospital, residential and community	Residential	Residential and hospital	Community
**Innovation (What did they do or change?)**	Established a 'vulnerable groups taskforce' made up primarily of fire dept employees to inspect nursing homes and run tests on staff,<br>Established isolation areas within nursing homes for residents who are CV positive and 'clearing zones' for new residents who do not have infection<br>Building a field hospital to treat CV positive patients who do not	Hospital physicians collaborating with GPs and community teams to deliver care directly in the home setting<br>Transfer of residents to the acute setting<br>Using technology to maintain contact with district nurses and GPs<br>General practice and community teams across the	Collaboration between local authorities, health board, Macmillan and Wales co-operative center to supply Ipads into residential and community settings enabling:<br>Piloting of a 'Attend Anywhere' project which allows for GPs to deliver a remote consultation service within care homes<br>Isolated patients in care homes and acute settings to communicate with	Expanded role and adaptation to PPE use as well as compensating for increased isolation of clients<br>Some voluntarily providing 24 care to older people in need of support<br>Delivering food parcels as well as inter-organisational support to cover staff shortage in face of increased demand

	require intensive care<br>Retired doctors and family doctors unable to practice recruited to run a telephone service<br>Medical students recruited to support an electronic reporting system and for emergency care and patient monitoring<br>Establishing corona teams consisting of physicians who will test and monitor patients in their homes	health boards increasing support to patients in care homes<br>Regular contact from GPs to care homes<br>Social services supporting care homes through financial support, advice and protective equipment	family but also physicians communicating with family too.	
**Social processes (How or why did they do it? Motivations, reasonings, resources, competencies)**	Commitment to their role and willingness to expand it to meet the challenge (retirees returning to work and med students working as ancillary).<br>Integration of acute services to the community—touring 'corona teams	Commitment to their role and willingness to expand it to meet the challenge<br>Integration of care home services with acute settings<br>Greater inter-professional collaboration and support from community care teams (GPs, PHNs) to support care homes<br>Expansion of role of social services	Adapting scope of practice to improve integration of services between community (GP) and residential settings<br>Improved information sharing and communication	Commitment to their role and a willingness to expand it to meet the challenge
**New ways of working (Outcome)**	Integration of acute and community services<br>Role expansion	Expanding scope of practice<br>Integration between acute, community and residential		Expansion of scope of practice and inter-organisational support.

Table s4. Extraction Table for Retrieved Newspaper Articles (*N* = 34) cont.

Title of article	**'A lot of People have Stepped Up and are Working Long Hours'; Adult Social Care Director Hails…**	**Care Home Workers' Week-Long Live-Ins with Residents**	**Bucks-Based Home Care Go 'above and beyond' for Clients and Employees**	**Care Home Workers on Battle they Face to Keep Residents Safe**
**Source**	Stoke The Sentinel	Eastern Daily Press	Bucks Free Press	Grimsby Telegraph
**Byline or Author(s)**	Kathie Mcinne	N/A	Kiera Gillies	Peter Craig
**Date of publication**	15.04.20	15.04.20	11.05.20	15.04.20
**Country of publication**	England	England	England	England
**Country of article focus**	England	England	England	England
**Health and social care personnel involved**	Doctors, carers	Residential care staff	Home Carers	Home carers
**Context for change (community, hospital, rehab or residential care)**	Community	Residential	Community	Community
**Innovation (What did they do or change?)**	Council staff redeployed into home carer roles—many of home are retraining to do the role	Week on week off living in the care home to prevent spread of virus (working in teams)<br>Expanding their scope of practice to cover ancillary services which have been withdrawn from the home (hairdressing, entertainment)<br>Using virtual tech to maintain contact between residents and family	Virtual meetings every week to deliver training and motivational talks from experts including:<br>Advice from a home economist on diet and meal planning<br>Podiatrists sharing videos and expertise to assist carers in footcare<br>Yoga classes<br>Ongoing support for carers through telephone calls and motivational speakers<br>Closed facebook group for carers to share photos and activities<br>An online care	Increased their role to include shopping, collecting medication and ensuring all of the needs of their clients (older people) are met during isolation from family

			monitoring system to provide oversight of quality and safety of care	
**Social processes (How or why did they do it? Motivations, reasonings, resources, competencies)**	Volunteering to be redeployed into carer role because that was where the challenge was—to support vulnerable people (esp older people) in the community and to transition people out of acute care settings.	Commitment to their role and to the residents	Role expansion (nutrition and podiatry) Online virtual community support for carers with a view to improving care quality	Commitment to their job and to their clients results in willingness to go above and beyond
**New ways of working (Outcome)**	Role expansion and redeployment	Role expansion	Role expansion and commitment Establishing a virtual community to provide support and skills acquisition	Expanded role

Table S5. Extraction Table for Retrieved Newspaper Articles (*N* = 34) cont.

**Title of Article**	**Elderly in Transfer to Udston Hospital**	**Front Line Lives: Care Home Staff on Working through Covid-19**	**Health and Social Care Coronavirus Pledge Given by NHS and North Lanarkshire Council; Working Whenever Possible as a Single Organisation, the Approach will be to Deliver and Manage many Health and Social Care Services Across Lanarkshire.**	**Heroes of Home Front**
**Source**	Winshaw Press	The National	Daily Record	The Sun (UK)
**Byline or Author(s)**	N/A	Karrin Goodwin	Ross Thomson	Lorraine Kelly
**Date of publication**	08.05.20	10.05.20	18.03.20	4.04.20
**Country of publication**	Scotland	Scotland	England	England
**Country of article focus**	Scotland	Scotland	England	England
**Health and social care personnel involved**	Consultant Geriatrician, allied health professionals, doctors, nurses	Social Carers workers (home carers, first responders, care home)	Social care and council workers.	Nursing home staff
**Context for change (community, hospital, rehab or residential care)**	Hospital	Residential & Community	Community	Residential (nursing home)
**Innovation (What did they do or change?)**	Reconfiguration in the care of older people moving ward our of acute hospital to a new setting.<br>Maintained same providing seven days clinical team	Reviewing information and guidance quickly for implementation in their daily work.<br>Taking on new roles—'fulfilling the role of district nurses'<br>Checking in on older people who previously received home care visits in their own time.<br>In residential setting celebrating birthdays and bringing residents outside to the garden during Covid19.	Three council social care staff working together to reorganise supports for older people<br>Improve communication and cooperation between staff.	Staff taking place of families personalising their care and establishing virtual communication for family.<br>Staff moving into the nursing home toc are for residents.

**Social processes (How or why did they do it? Motivations, reasonings, resources, competencies)**	Commitment to their role and willingness to adapt it to provide ongoing care to older people.	Role expansion<br>Commitment of emotional support for patients.	Inter-professional relationships leading to good 'team spirit' rising to the challenge	Role expansion and compensation<br>Commitment to social and emotional support for patients
**New ways of working (Outcome)**	Physical restructuring	Role expansion<br>Learning and implementing guidance on news ways of working. Social and emotional communication	Improved information sharing and communication	Role expansion<br>Social and emotional communication

Table S6. Extraction Table for Retrieved Newspaper Articles (*N* = 34) cont.

**Title of article**	**Heroes of the Corona Crisis**	**Heroic Staff to go into 24/7 Quarantine for a MONTH with OAPs**	**How are our Pharmacists Coping with Covid Crisis?; The Coronavirus has Transformed the Way Chemists are Working. Here they Speak about how they, their Staff and their Customers are Dealing with the New Situation**	**How Work has Changed for Carers during Health Crisis**
**Source**	Scottish Daily Mail	Scottish Daily Mail	Irish Times	South Wales Echo
**Byline or Author(s)**	NA	Katherine Sutherland	Danielle Barron	Richard Youle
**Date of publication**	14.04.20	12/4/2020	21.04.20	18.04.20
**Country of publication**	Scotland	Scotland	Ireland	Wales
**Country of article focus**	Scotland	Scotland	Ireland	Wales
**Health and social care personnel involved**	Healthcare teams	Staff in a care home (chefs, assistants nurses, manager)	Community Pharmacists & GP'S	Experience of formal carers
**Context for change (community, hospital, rehab or residential care)**	Hospital	Residential (nursing home)	Community	Community & Residential setting
**Innovation (What did they do or change?)**	Taking on new roles-e.g. consultant plastic surgeon took the role of a nurse in an ICU unit.	Activities such as baking art and singalongs organised to compensate for visitor restrictions.	Reorganisation of services to provide care to older patients.<br>Arranging deliveries of prescriptions for some older people who are	Taking on new information around PPE and adapting her work due to Covid 19.<br>Staff personalising the care

	Regular communication, briefings and teamwork between disciplines	Staff created a space outside and a booking system for family residents to visit residents safely. Sleeping in the care home to limit exposure at home. Interrelationships between carer/nurse and the patient—'taking the place of family'	cocooning. Accepting electronic prescriptions from GP's via one system (previously paper based).	of residents doing hair and nails of residents to keep spirits up.
**Social processes (How or why did they do it? Motivations, reasonings, resources, competencies)**	Commitment to their role and willingness to expand it to meet the challenge Recognition of the importance of communication and finding ways to communicate and share information.	Commitment to the role—willingness to expand it and take on more hours to meet the challenge Recognition of the importance of social and emotional connection in the absence of physical contact	Inter-professional relationships Using technology to improve information sharing and communication.	Role expansion Commitment to social and emotional support for patients
**New ways of working (Outcome)**	Role expansion and improved info sharing/communication	Role expansion Social and emotional communication Physical restructuring	Improved information sharing and communication. Role expansion.	Role expansion Social and emotional communication

Table S7. Extraction Table for Retrieved Newspaper Articles (*N* = 34) cont.

**Title of article**	Inside the Mater's war on Covid-19; If the beds run out, we'll drop special medical pods in the car park, hospital chief executive Alan Sharp tells Maeve Sheehan	Medicine and food parcels are on the way	Meet and greet service when leaving hospital; Door-to-door taxis for vulnerable patients	Meet NHS staff using skills to help in frontline: they've stepped away from current roles
**Source**	Sunday Independent	Paisley Daily Express	Stoke the Sentinel	Leicester Mercury
**Byline or Author(s)**	Maeve Sheehan	Steph Brawn	Kathie McInnes	Staff reporter
**Date of publication**	22.03.20	4/4/2020	09.05.20	01.05.20
**Country of publication**	Ireland	Scotland	England	England
**Country of article focus**	Ireland	Scotland	England	England
**Health and social care personnel involved**	HSCP's (including CEO)	Health and social care	Health and social care	Nurses
**Context for change (community, hospital, rehab or residential care)**	Hospital and community	Community	Hospital to home	Statutory bodies (NHS England and NHS Improvement) and hospitals
**Innovation (What did they do or change?)**	Taking on new roles within the acute hospital.<br>Regular communication, briefings with partners in step down hospitals ensuring older people were transferred.<br>Sharing advice and info across sites.	Taking on new roles-developed a medicine delivery service for older people in the community.<br>Role expansion for teams in other areas in the council doing welfare and food parcel deliveries for older people.<br>Sharing advice and info across the council.	Taking on new roles-developed a transport from hospital to home system with local taxi companies for older people.<br>Provided ongoing welfare checks.<br>Increased social care supports to seven days a week.	Voluntary redeployment of nurses working in senior managerial/strategic roles in NHS England and NHS Improvement to support front-line teams working in hospitals<br>Transfer from head office to increase capacity on front line and share expertise and knowledge.
**Social processes (How or why did they do it? Motivations,**	Commitment to their role and willingness to expand it to meet the challenge<br>Recognition of the importance of	Interrelationships between council staff.<br>Commitment to the role—willingness to expand it and take	Inter-professional relationships leading to good 'team spirit' rising to the challenge	Recognition that hospitals required additional resources and expertise<br>Knowledge sharing—bringing skills and expertise with them to assist

**reasonings, resources, competencies)**	communication and finding ways to communicate and share information.	on more hours to meet the challenge<br>Willingness to share expertise and knowledge	Development of social relationship with older people - regularly 'checking in' on them.	hospital staff.<br>Willingness to share knowledge/expertise<br>Communication<br>Commitment to working with colleagues in hospitals to ensure patients receive the care that they need during C-19 pandemic<br>Willingness—role change / role expansion / expanded scope of practice.<br>Adaptability, flexibility to take on new roles
**New ways of working (Outcome)**	Role expansion and improved info sharing/communication	Role expansion, social restructuring and improved info sharing/communication	Role expansion<br>Improved info sharing and communication	Reorganisation of human resources across organisations<br>New knowledge sharing<br>Flexibility in scope of practice.

Table S8. Extraction Table for Retrieved Newspaper Articles (*N* = 34) cont.

**Title of article**	**Undervalued care workers continue to show just how vital they are in fight against coronavirus; Hard working and selfless carers are unsung heroes of this crisis**	**These elderly people are so vulnerable—it's our job to look after them; with the Grand National cancelled, the ECHO has launched You Bet We Care, to encourage readers to donate their stake to Liverpool City Region Metro Mayor Steve Rotheram's £1m fundraising campaign—lRC Cares. Here's an example of how the cash could be used …**	**Picture of Selflessness**	**MP praise for coronavirus lifeline**
**Source**	Liverpool Echo	Liverpool Echo	Daily Mail (London)	Ayr Advertiser
**Byline or Author(s)**	Jenny Kirkham	Cheryl Mullin	Helen Weathers	Ryan Thorn
**Date of publication**	13.04.20	02 04 20	04.04.20	09.04.20
**Country of publication**	England	England	England	Scotland
**Country of article focus**	England	England	England	Scotland
**Health and social care personnel involved**	HSCPs (carers)	Age Action staff and community volunteers	HSCPs (carers, chef and kitchen and domestic assistants) and deputy manager	Staff and volunteers at South Ayrshire Lifeline (a caring call centre set up by Voluntary South Ayrshire (VASA)
**Context for change (community, hospital, rehab or residential care)**	Community (nursing home)	Community (people living at home)	Residential (nursing home)	Community (people living at home)
**Innovation (What did they do or change?)**	Care staff sleeping in the care home to limit risk of exposure to the residents	Collaboration between Age Action and community volunteers to provide additional supports to older people (cocooning) in the community:<br>Financial assistance<br>Food packages distribution<br>Prescription collection<br>Well-being calls to assess level of need and	Staff sleeping in the care home to limit exposure to the residents<br>Working longer hours without additional pay	Call centre collaboration with volunteers to provide additional assistance to older persons in the community:<br>Wellbeing calls to provide emotional

		regular check-in calls, if needed.<br>New team working—Age Action matching volunteer to older person in need.		support (listening ear)<br>Food deliveries,<br>Prescription collection support for people with sight or hearing impairment and hearing aid batteries by post<br>New team working—call centre and volunteers
**Social processes (How or why did they do it? Motivations, reasonings, resources, competencies)**	Dedication/commitment to role—flexibility/adaptability to change to meet the challenge.<br>Restructuring working hours—carers sleeping on premises<br>Willingness to change working conditions	Recognition of the emotional and physical needs of older people who may not have other means of support during cocooning.<br>New team work—Age Action and community volunteers working together to provide support to older people.<br>Communication and information sharing<br>Developing social relationships with older persons	Dedication/commitment to —flexibility/adaptability willingness to take on longer hours to protect residents<br>Restructure working hours—carers sleeping on premises<br>Willingness to change working conditions	Recognition of the emotional and physical needs of older people who don't have other means of support during cocooning.<br>New team work—between call centre staff and volunteers to provide support to older people.<br>Communication and information sharing<br>Developing social relationships with older persons
**New ways of working (Outcome)**	Role—restructuring of working hours<br>Change of working conditions	Role expansion<br>New ways of working—new team formation / working beyond normal team boundary	Role—restructuring of working hours<br>Change in working conditions	Role expansion<br>New ways of working—new team formation / working beyond normal team boundary

Table S9. Extraction Table for Retrieved Newspaper Articles (*N* = 34) cont.

**Title of article**	**“Step up to the Mark”: Council Transforms Social Care Services to Look after the Elderly**	**Pulling Together to Help the Frontline**	**We’re all in this together. Links between Private and Public Healthcare Forged in the Pandemic will Live on, says Spire Chief Justin Ash**	**New Covid-19 Rehabilitation Hospital to open in former TB Wards**
**Source**	Craven Herald	South Wales Echo	The Sunday Times (London)	Sunday Independent
**Byline or Author(s)**	Herald reporter	Aamir Mohammed	Sabah Meddings	Maeve Sheehan
**Date of publication**	10.4.2020	20.04.20	10.05.20	03.05.20
**Country of publication**	England	Wales	England	Ireland
**Country of article focus**	England	Wales	England	Ireland
**Health and social care personnel involved**	Front line social care teams (inc carers), hospital doctors, GPs, local authority staff, community health staff	Frontline health workers—hospital and community Community volunteers	Doctors and nurses	Geriatrician, staff of nursing home, hospital staff and IEHG staff
**Context for change (community, hospital, rehab or residential care)**	Hospital, community (nursing homes) (homes of older people)	Hospital and community (primary care, nursing homes, palliative care and people’s homes)	Private and NHS hospitals	Hospital and community (nursing home)
**Innovation (What did they do or change?)**	Council transformed its social care service to improve capacity of NHS in preparation of coronavirus peak.<br>Front line social care team (council employees), some whom redeployed, worked with hospital staff to expedite discharge of older people from hospital to appropriate community setting (nursing home or their own home)<br>Front line social care team (council) extended hours of service in community (to 7 days 8 am-8pm) and work with primary health care (GPs and other community staff) to provide emergency support and treatment for	Community fundraising for provision of PPE to GP practices, nursing homes and palliative care services, and respirators for local ICU.<br>GP consultations changed to phone and video consultations. Ace to -face consultations only if necessary.<br>Redeployment of orthopedic surgeons in hospital to assist in care of	New contractual relationship between NHS and private healthcare group to increase capacity (additional ICU beds) and continue treatment for patients requiring elective surgery, treatment of cancer patients and midwifery services.<br>Redeployment of NHS doctors and nurses to private hospitals to provide services alongside those employed by private hospitals	Transformed former TB unit on site of nursing home to a rehabilitation post-covid ward for people of all ages<br>Transfer of staff from hospital to work on new ward and work in nursing home (re latter to compensate for shortages of staff due to covid)<br>Integration of laboratory medicine into nursing home setting—hospital lab resources used for testing staff

	community based people with covid, (No reference to age)<br>Council working with nursing homes to provide support and guidance—including introduction of segregated areas in nursing homes.	covid patients.<br>Segregation of covid and non-covid patients in hospital by introduction of separate zones.		and residents at nursing home to identify covid and improve risk management.<br>Integration of other hospital services (non specified) with nursing home—residents given greater access to services.
**Social processes (How or why did they do it? Motivations, reasonings, resources, competencies)**	Willingness to extend role (scope of practice); willingness to take on new role.<br>Increased integration between hospital and council employees (front-line) in respect of discharging patients. Working beyond normal team boundaries.<br>Increased communication and information sharing.<br>Recognition of additional capacity required by NHS hospitals to treat covid patients.<br>Willingness to work longer hours—dedication/commitment to role<br>Willingness to work outside normal team boundary in the community<br>Increased teamwork, communication and information sharing.<br>Willingness to share expertise and knowledge with nursing homes.<br>Willingness of nursing home to adapt to new physical infrastructure and to adapt to advice provided for mitigation of risk of contagion to residents and staff<br>Knowledge sharing<br>Restructuring physical infrastructure to provide segregated zones	Fundraising: Recognition of need to reduce risk to frontline workers and patients. Willingness to fundraise.<br>GP service—recognition of need to reduce risk of transmission of virus. Use of technology for consultations -adapting pathway of care.<br>New means of communication.<br>Face-to-face consultations where necessary.<br>Willingness to change systems of work.<br>Orthopedic surgeon redeployment—recognition of need to maximise available resources.<br>Willingness to work in different team / specialty.<br>Role expansion, role change.<br>Recognition that risk management requires minimal exposure by non-	Ensuring sufficient capacity in health service to treat covid-19 patients<br>Recognition of importance of continuing regular services—ensuring elective surgery, cancer treatment and midwifery care can continue<br>Reorganisation—transfer of some services to new location and transfer of staff<br>Redeployment of NHS doctors and nurses—willingness to work in new physical environment, willingness to adapt during crisis, willingness to work in new team.<br>Partnership approach between NHS and private health care organisation.	Integration of services across hospital, temporary ward and nursing home<br>Diagnostic test—may reduce isolation period and increase capacity of workforce.<br>Increased collaboration between nursing home, hospital and IEHG.<br>Transfer of staff from hospital setting<br>Recognition of need for additional capacity to treat covid rehab patients and recognition of need to compensate for acute staff shortages due to covid at nursing home.<br>Role expansion—willingness to work across different orgnaistions<br>Willingness to work beyond normal team boundary<br>Willingness to work in new physical settings and new teams

		covid patients to those with covid. Willingness to change physical infrastructure.<br>Forward planning, communication, staff adapting to new ways of working.<br>Willingness to change pathways / re-assign physical spaces within hospital.	
**New ways of working (Outcome)**	Increased integrated care across hospital and community boundary and within the community<br>Reorganisation of services to meet challenge (working conditions and working in extended teams)<br>Reorganisation of physical infrastructure (nursing homes)	Adapted pathways of care<br>New ways of communication with patients.<br>Role expansion<br>Working outside of normal team boundary, working within new teams	Physical reorganisation of services—transfer of services to the private sector<br>Reorganisation of staff—transfer of staff to new location<br>Patients cared for in private hospital, care funded by NHS and provided by mix of NHS and private hospitals staff.<br>Public and private partnership sharing NHS human resources (staff)

Table S10. Extraction Table for Retrieved Newspaper Articles ($N = 34$) cont.

Title of Article	"We haven't had time to grieve" Care Homes Struggle as Covid-19 Deaths Rise. UK's Largest Provider Says 10% of All Staff are Self-Isolating as Lack of PPE Testing takes Toll across the Sector	Team Effort gets Tablet Computers to most Vulnerable
**Source**	The Guardian (London)	Wirral Globe
**Byline or Author(s)**	Robert Booth Social Affairs Correspondent	George Morgan
**Date of publication**	09.04.20	01.05.20
**Country of publication**	England	England
**Country of article focus**	England	England
**Health and social care personnel involved**	Staff in nursing homes and management teams of MLA (charity providing nursing home care)	Coordinated approach between business sector, voluntary sector, Wirral Council and Age Action UK.
**Context for change (community, hospital, rehab or residential care)**	Community (nursing home)	Community (vulnerable people including people living alone and carers of people with dementia)
**Innovation (What did they do or change?)**	Staff facilitated communication between dying and their family and friends in the absence of physical contact (saying goodbye through windows, passing messages through carers, telephone, cards)<br>Staff moved into facility in order to minimise their own and residents' exposure to virus. Reduce risk of contagion.<br>Virtual management team meetings across group using video conferencing to assist and provide practical support to each. Triage system to determine priority of response.	Distribution of tablets to people classed as vulnerable to:<br>enable them to maintain contact with friends, family etc.<br>to access mindfulness and relaxation techniques on line to help combat mental strain of isolation<br>to access exercise programmes online<br>to access mental stimulation through online games/quizzes<br>Developing of user training package.
**Social processes (How or why did they do it? Motivations, reasonings, resources, competencies)**	Recognition of emotional needs of residents and their families when resident dying.<br>Facilitation of communication between resident and loved ones in absence of physical contact.<br>Adapting and finding new means of communication.<br>Dedication / commitment to role. Role expansion. Willing to sleep at nursing home to minimise risk of resident's exposure to virus.<br>Mutual recognition of challenges to each nursing home in group—team approach to problem solving, triaging on basis of most urgent needs, sharing	Coordinated approach between Age UK, Vodafone, Wirral Council and voluntary charitable groups.<br>Recognition of negative effects of social isolation on vulnerable people<br>Recognition of educational needs—development of training package<br>Communication/information sharing

**New ways of working (Outcome)**	resources. Willingness to share knowledge and resources. New ways of communicating with each other.<br>New means of communication<br>Role expansion<br>Working outside usual scope of practice—sharing knowledge and resources—mutual benefit of each nursing home	Teamwork—working beyond normal team boundary.

END
